# Patterns of Theta Activity in Limbic Anxiety Circuit Preceding Exploratory Behavior in Approach-Avoidance Conflict

**DOI:** 10.3389/fnbeh.2016.00171

**Published:** 2016-09-22

**Authors:** Luis R. Jacinto, João J. Cerqueira, Nuno Sousa

**Affiliations:** ^1^Life and Health Sciences Research Institute, University of MinhoBraga, Portugal; ^2^ICVS/3B's – PT Government Associate LaboratoryBraga/Guimarães, Portugal

**Keywords:** anxiety, stress, local field potentials, ventral hippocampus, amygdala, prefrontal cortex

## Abstract

Theta oscillations within the hippocampus-amygdala-medial prefrontal cortex (HPC-AMY-mPFC) circuit have been consistently implicated in the regulation of anxiety behaviors, including risk-assessment. To study if theta activity during risk-assessment was correlated with exploratory behavior in an approach/avoidance paradigm we recorded simultaneous local field potentials from this circuit in rats exploring the elevated-plus maze (EPM). Opposing patterns of power variations in the ventral hippocampus (vHPC), basolateral amygdala (BLA), and prelimbic (PrL) mPFC, but not in the dorsal hippocampus (dHPC), during exploratory risk-assessment of the open arms preceded further exploration of the open arms or retreat back to the safer closed arms. The same patterns of theta power variations in the HPC-BLA-mPFC(PrL) circuit were also displayed by animals submitted to chronic unpredictable stress protocol known to induce an anxious state. Diverging patterns of vHPC-mPFC(PrL) theta coherence were also significantly correlated with forthcoming approach or avoidance behavior in the conflict situation in both controls and stressed animals; interestingly, vHPC-BLA, and BLA-mPFC(PrL) theta coherence correlated with future behavior only in stressed animals, underlying the pivotal role of the amygdala on the stress response.

## Introduction

Emotional disorders are prevalent in western societies. WHO data shows that disorders within the anxiety spectrum target over 15% of the western population (Kessler et al., 2001). State anxiety arises from unexpected features in the environment and is classically viewed as an evolutional survival response. This transitory state prepares the individual to eventual harmful encounters in contexts where the presence of any immediate discrete threat is uncertain. It is usually characterized by heightened arousal and vigilance (Blanchard et al., 1991; Rodgers et al., 1997; Davis et al., 2010). However, anxiety can also arise from competing motivations when a decision has to be made in an environment perceived as potentially aversive and/or where reward is uncertain (Gray and McNaughton, 2003; Bailey and Crawley, 2009). This view attributes a critical role to decision-making in the anxiety response and also explains why the most commonly used anxiety tests for animals rely on unconditioned responses to competing innate appetitive and aversive motivations (Davis et al., 2010). Central to the process of resolving the conflict of competing motivations in an anxiogenic context is the concept of risk assessment. This defensive behavior is part of the constellation of anxiety-like behaviors and is the process through which a potentially aversive environment/stimulus can be cautiously explored/approached allowing the gathering of information while heightened arousal is still maintained (Blanchard et al., 1991, 2011; Rodgers et al., 1997; Blanchard, 2003; Cryan and Holmes, 2005). For some authors, this is precisely what defines anxiety and what separates it from a fear response usually involving a flight or fight response to a clearly present threat (Gray and McNaughton, 2003; Blanchard et al., 2011). Risk assessment is therefore one of the most important behaviors of the anxiety response as it allows contextual information encoding/processing and guides decision-making in an anxiety-provoking environment toward approach or avoidance of the potentially aversive stimuli/context, ultimately leading a return to basal behavior (Blanchard et al., 1991; Rodgers et al., 1997; Blanchard, 2003; Cryan and Holmes, 2005; Blanchard et al., 2011).

The circuit formed by the ventral hippocampus (vHPC), the medial prefrontal cortex (mPFC), and the amygdala (AMY) has a preponderant role in emotional behavior. In recent years, several studies, including from our lab, have shown that activity within this circuit is critical for the expression of anxiety-related behavior (Adhikari et al., 2010; Jacinto et al., 2013). Anatomically, the vHPC is strongly connected with the mPFC and AMY, usually in a reciprocal way (Pitkänen et al., 2000; Ishikawa and Nakamura, 2003; Orsini et al., 2011), further reinforcing the idea of a unified circuit with a preponderant role in emotional responses. Theta oscillations, in particular, can provide temporal synchronization within the vHPC-AMY-mPFC circuit (Lesting et al., 2011) and, thus, have been implicated in the modulation of emotional behaviors, including anxiety (Adhikari et al., 2010; Jacinto et al., 2013) and fear (Seidenbecher et al., 2003; Popa et al., 2010; Lesting et al., 2011).

Chronic exposure to stress can impact trait anxiety by increasing the sensitivity to aversive stimuli (Pêgo et al., 2008; Sousa, 2016). For example, individuals with post-traumatic stress disorder tend to show a persistently higher sensitivity to anxiety-provoking stimuli and therefore display disproportionate and long-sustained anxiety responses to those stimuli (Gorman, 2002). Chronically stressed animals also display increased aversion across various contexts (Sousa, 2016). Interestingly, stress exposure is known to impact the activity of the vHPC and BLA (Rainnie et al., 2004; Kavushansky and Richter-Levin, 2006; Maggio and Segal, 2009; Oliveira et al., 2013; Pinto et al., 2015) including in an anxiogenic context (Jacinto et al., 2013).

Surprisingly, no previous study has assessed the neural computations that occur during conflict decision-making. Thus, herein, we recorded local field potentials (LFP) in the vHPC, dorsal hippocampus (dHPC), basolateral amygdala (BLA), and pre-limbic (PL) region of the mPFC in rats freely behaving in the EPM; in particular, our analysis focused on theta power and theta coherence variations in the initiation of exploration of the open arms, the so called exploratory risk-assessment, as this is the critical point of decision in the exploration/avoidance conflict posed by the EPM. In addition, we assessed whether the same readouts would be of value in rats exposed to a chronic unpredictable stress (CUS) protocol known to induce anxious behavior. Our goal was to observe if differential activity or synchronization routes within the vHPC-BLA-mPFC(PrL) circuit could underlie the different behaviors of controls and stressed animals in the EPM.

## Results

### Behavior in the EPM

When entering the open arms (mean number of entries: 10.50 ± 2.11), control animals displayed risk-assessment behavior (head dips and front paws' entries; mean time spent on risk-assessment entries: 5.33 ± 0.51 s). In 20% of the cases, this behavior was followed by a complete entry into the open arm (approach action), and on the remainder (80% of the cases) it resulted in a retreat to the closed arms (avoidance action). In contrast, the majority of closed arm entries were fast full body transitions without any preceding risk-assessment activity (mean number of closed arm entries: 10.66 ± 2.16). Time spent in the open arms was on average ~30% of the total time of the test (mean open arm exploration time: 101.00 ± 15.30).

### Theta activity in the vHPC-BLA-mPFC(PrL) circuit predicts exploratory outcome of risk-assessment behavior

**Figure 1:**
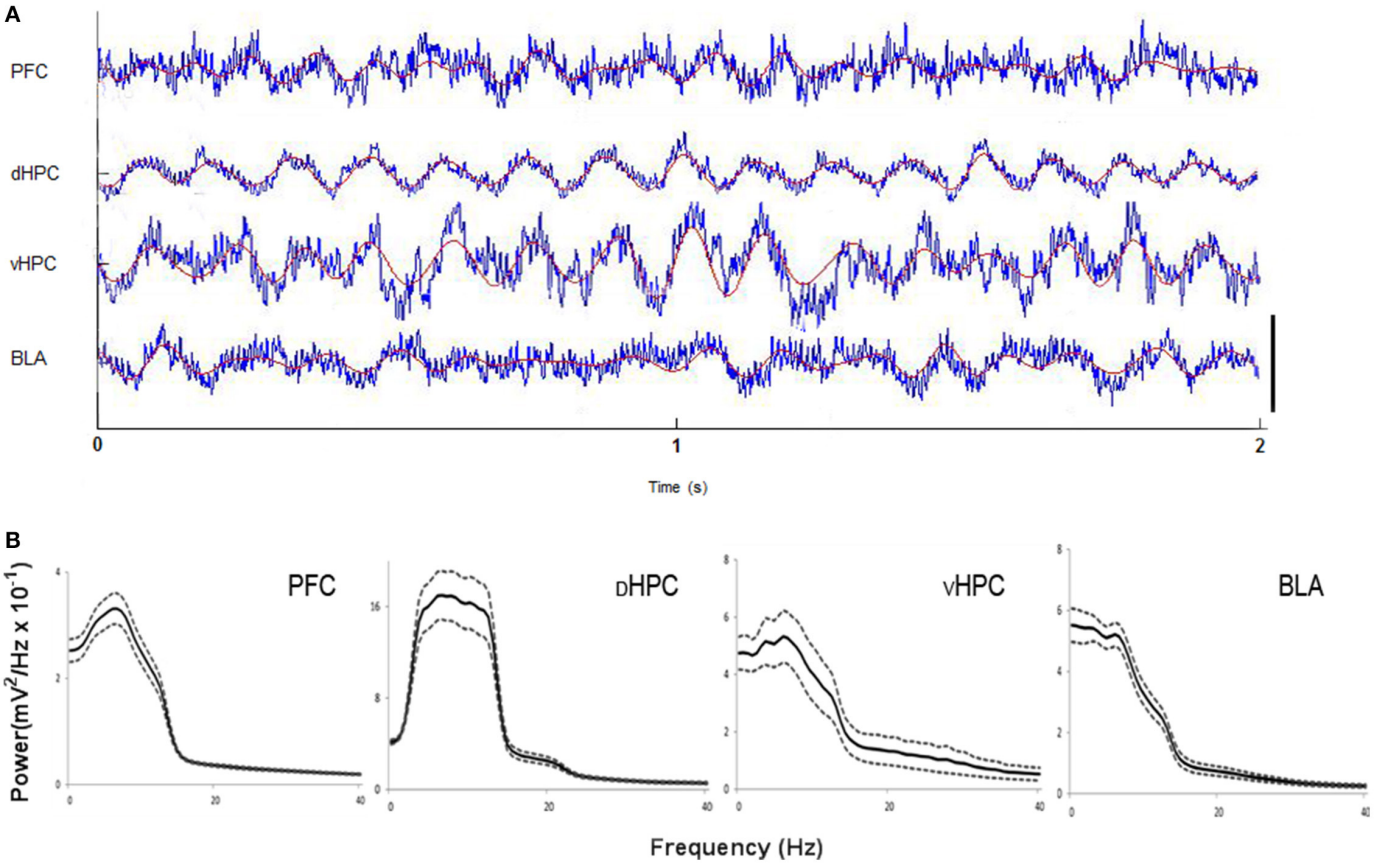
**(A)** Representative traces from local field potentials simultaneously recorded from the medial prefrontal cortex (mPFC(PrL)), dorsal hippocampus (dHPC), ventral hippocampus (vHPC) and basolateral amygdala (BLA) in one rat performing the Elevated-Plus Maze (EPM) test. Raw traces are plotted in blue and filtered theta traces (5–12 Hz) are overlayed in red. Presented segment duration is 2 s. Voltage scale (bottom right) is −0.2 to 0.2 mV for mPFC(PrL), vHPC and BLA; and −0.4 to 0.4 mV for dHPC. **(B)** Power spectra for mPFC(PrL), dHPC, vHPC, and BLA. Spectra are average of multitaper spectrum estimates for all animals (*n* = 10) during EPM exploration. Dotted lines are ± s.e.m.

Local field potentials were recorded by electrodes positioned in the dHPC, vHPC, BLA and mPFC(PrL) (Figure S1) in freely behaving rats during EPM performance. As expected, during exploratory behavior robust theta oscillations (5–12 Hz) were observed in LFPs recorded from the dHPC (McFarland et al., 1975; Hinman et al., 2011) and, with equal robustness, but lower magnitude, in the vHPC, mPFC, and BLA (Adhikari et al., 2010; Royer et al., 2010; Lesting et al., 2011; Patel et al., 2012; Schmidt et al., 2013). Figure 1 shows representative traces of simultaneously recorded local field potentials during risk-assessment from the dHPC, vHPC, BLA and mPFC(PrL) and respective power spectra, with theta activity being visible in all brain areas.

Variation of theta power between the period immediately preceding the risk-assessment period (0.5 s; baseline)—when the animal is in the center region of the EPM—and the first 1.5 s of risk-assessment behavior in the open arms was calculated as described in the methods section. This period was chosen because we were especially interested in observing the changes during the period in which the animals displayed risk-assessment behavior that preceded the actions to either fully enter (approach) or retreat (avoid) from the open arm. Of notice, all risk-assessment behaviors lasted at least 1.5 s (more than half of them lasting between 1.5 and 2.0 s). The remaining time windows (in the cases that the exploratory period lasted more than 1.5 s) were also analyzed. The same theta activity trends described below for the 1.5 s windows were generally maintained throughout that period (data not shown) which leads us to believe that the state anxiety signal is set in this initial period and is of relevance to the exploratory behavior in this context. Risk-assessment periods were then divided into future approach or avoidance actions, as previously described. Baseline theta power, which corresponded to activity in the center of the EPM before open arm entry, also did not differ when the baseline of future approach and avoidance actions were compared, for all brain areas (Figure S2).

**Figure 2:**
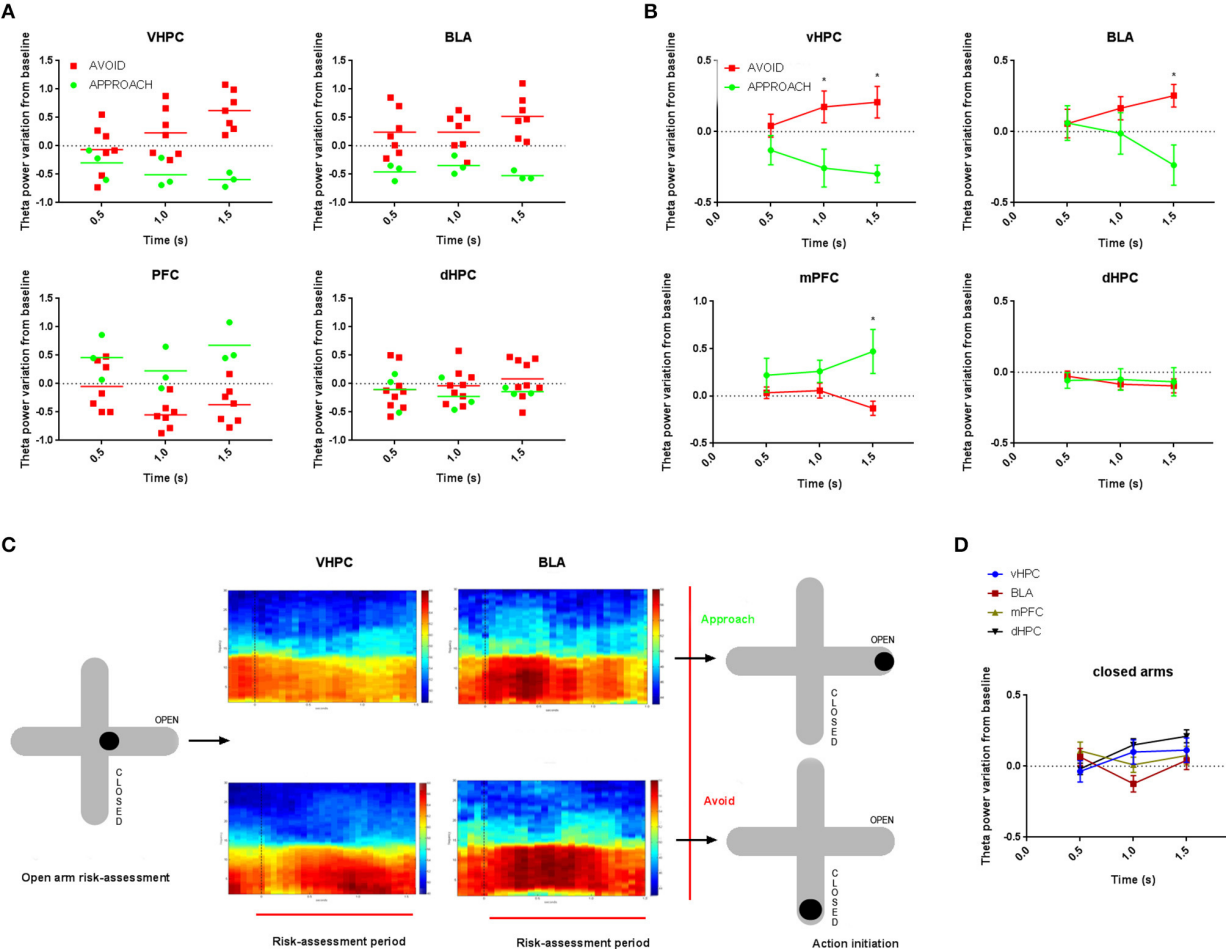
**Comparative time evolution of theta power variation from baseline in vHPC, BLA, mPFC(PrL) and dHPC during open arms risk-assessment preceding approach and avoidance actions (A) for one representative control animal; (B) and averaged for all actions for all control animals**. Baseline corresponds to the 0.5 s preceding the exploratory risk-assessment open arm entry. Data was averaged across animals for each time point according to the subsequent action (approach or avoid). **(C)** Representative average vHPC (left) and BLA (right) spectrograms during open arm risk-assessment preceding approach (top) and avoidance (bottom) actions for one control animal. The spectrogram for each area depicts power in the 0.5–30Hz range during risk-assessment period (0.0 to 1.5 s) and respective baseline (−0.5 to 0.0 s). Dotted line, at 0.0 s, marks the beginning of the exploratory risk-assessment period. **(D)** Comparative time evolution of mean average theta power variation from baseline following closed arm entry for all control animals in vHPC, BLA, mPFC(PrL), and dHPC. Data was averaged across animals for each time. ^*^p < 0.05 for unpaired Wilcoxon rank sum test comparison of average theta power variation between activity preceding approach and avoidance actions. Error bars, ± sem.

The variation of vHPC theta power during initial 1.5 s of risk-assessment exploration in respect to the 0.5 s preceding the risk-assessment period was remarkably different between subsequent approach and avoidance actions. While approach behaviors were preceded by a decrease in vHPC theta power following the risk-assessment period, the opposite was observed before avoidance behaviors (*p* < 0.05 for each significant *post-hoc* pairwise comparison between approach and avoid at 1.0 and 1.5 s; Figures 2A,B). In the BLA, theta power variation presented a similar, although slightly delayed, profile, with a clear difference between risk-assessment periods previous to approach and avoidance actions (*p* < 0.05 for *post-hoc* pairwise comparison at 1.5 s; Figures 2A,B). These results show that vHPC and BLA theta power increases during exploratory risk-assessment of the open arms that precede the action of withdrawing from them (avoidance), whereas fully entering the open arms (approach) is preceded by theta power decreases in the same regions. Figure 2C also shows an example of average spectrograms for vHPC and BLA of all risk-assessment periods preceding both approach and avoidance actions of one rat.

Interestingly, in the mPFC(PrL), theta power during the initial open arm exploration seemed to vary in the opposite direction, with a significant increase preceding approach behaviors (*p* < 0.05 for *post-hoc* pairwise comparison at 1.5 s; Figures 2A,B). dHPC theta power did not present any significant variation in respect to the baseline period nor between risk-assessment periods preceding approach or avoidance behaviors (Figures 2A,B).

Although, there were no apparent risk-assessment behaviors proceeding closed arm entries (all of which were fast full body transitions) we analyzed theta power variation following full closed arm entries in respect to the 0.5 s period immediately preceding them (baseline). Curiously, vHPC theta power variation from baseline during the first 1.5 s of closed arm entries was similar to that occurring before avoidance entries in the open arms, albeit with lower mean magnitude (Figure 2D). On the contrary, BLA and mPFC(PrL) theta power did not vary during closed arm entry (Figure 2D), while dHPC theta power steadily increased in respect to baseline (Figure 2D).

**Figure 3:**
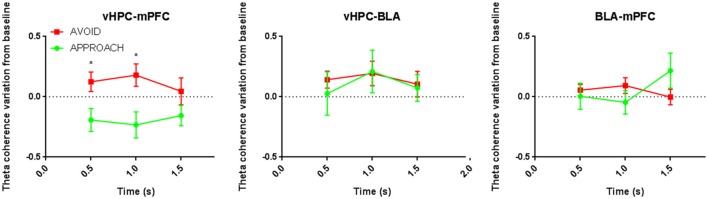
**Comparative time evolution of mean theta coherence variation from baseline during open arms risk-assessment preceding approach and avoidance actions for all control animals in vHPC-mPFC(PrL) (left), vHPC-BLA (middle), and BLA-mPFC(PrL) (bottom) brain areas' pairs**. ^*^p < 0.05 for unpaired Wilcoxon rank sum test comparison of average theta coherence variation between activity preceding approach and avoidance subsequent actions. Error bars, ± sem.

We then analyzed theta coherence between the regions that displayed differences in theta power during risk-assessment of the open arms preceding approach and avoidance actions. vHPC-mPFC(PrL) theta coherence varied in opposite directions immediately before approach and avoidance actions (*p* < 0.05 for each significant *post-hoc* pairwise comparison at 0.5 and 1.0 s; Figure 3), mimicking theta power variation in the vHPC. In contrast, vHPC-BLA and BLA-mPFC(PrL) theta coherence variations during risk-assessment were similar when preceding both approach and avoidance actions (Figure 3).

### Relevance of theta power activity in an animal model of hyperanxiety

**Figure 4:**
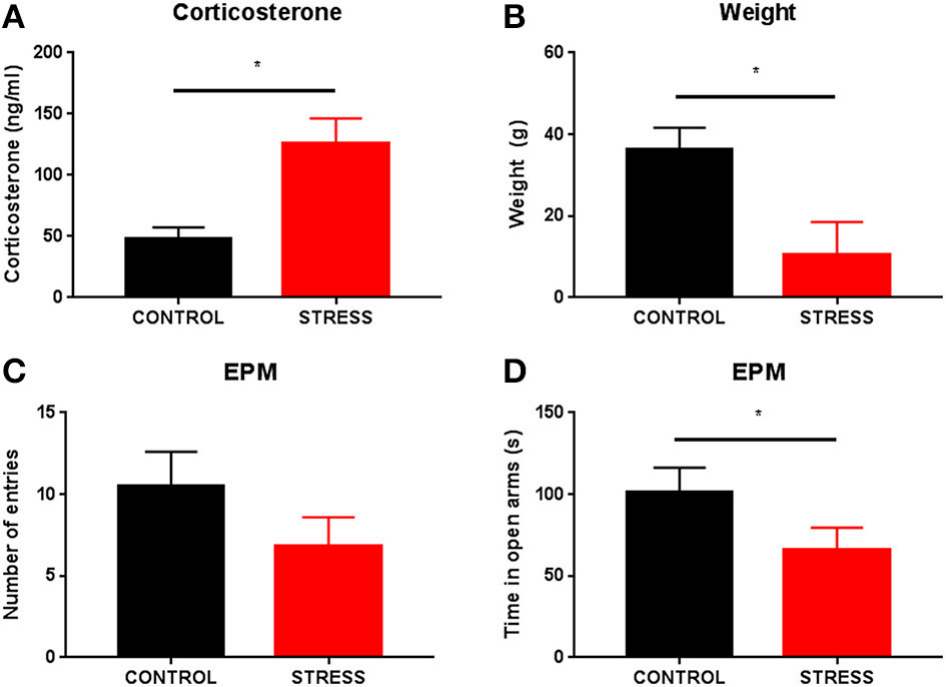
**Efficacy of the stress protocol and EPM behavior of stressed animals**. Comparison of serum corticosterone levels **(A)** and body weight gain between the beginning and ending of the stress protocol **(B)** between control and stressed animals. Comparison of anxiety-like measures in the EPM between control and stressed animals: **(C)** number of open arm risk-assessment entries and **(D)** time spent exploring the open arms. ^*^p < 0.05 for unpaired Wilcoxon rank sum test comparison of serum corticosterone levels, body weight gain, time spent exploring the open arms and number of open arm entries between control and stressed animals. Error bars, ± sem.

To verify whether the above-described variations in theta power were also observed in a validated animal model of anxiety, we exposed an additional group of animals to a 21-day chronic unpredictable stress (CUS) protocol previous to the EPM test (see methods). Stressed animals, when compared with controls, presented higher serum corticosterone levels (control: 48.00 ± 9.17 ng/mL vs. stress: 126.40 ± 19.85 ng/mL; *p* < 0.05; Figure 4A) and reduced body weight gain between the beginning and ending of the stress protocol (control: 36.40 ± 5.20 g vs. stress: 10.40 ± 8.13 g; *p* < 0.05; Figure 4B), thus confirming the biological efficacy of stress exposure.

In the EPM, stressed animals tended to enter the open arms less frequently than controls spending significantly less time exploring them when compared with controls (mean number of open arm entries: control 10.50 ± 2.10 vs. stress 6.8 ± 1.80, *p* = 0.22; mean time exploring open arms: control 101.00 ± 15.30 vs. stress 66.00 ± 13.60, *p* < 0.05, Figures 4C,D respectively).

**Figure 5:**
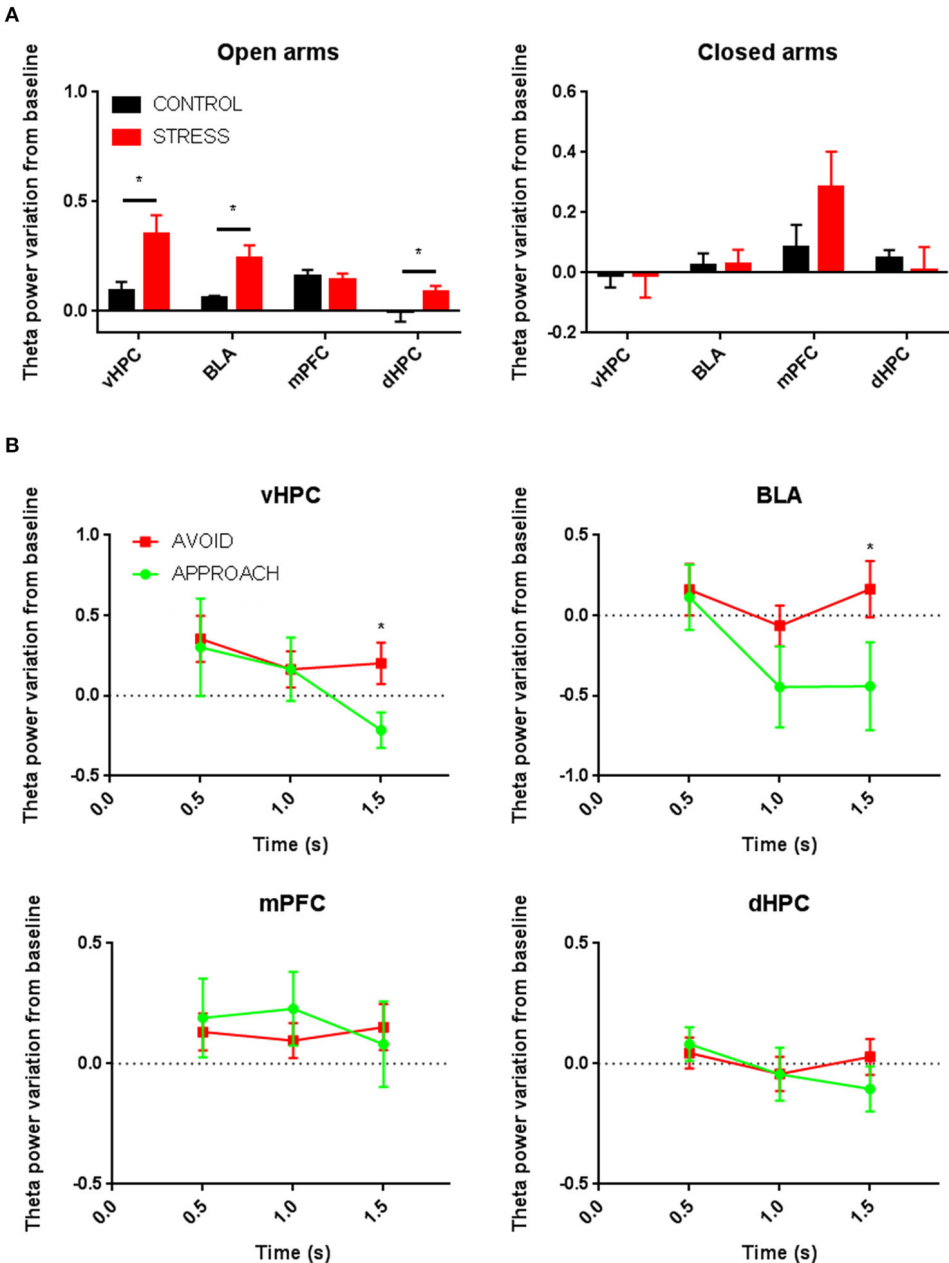
**(A)** Comparative mean theta power variation from baseline between control and stressed animals immediately following open arms risk-assessment entry regardless of subsequent approach and avoidance actions (left); and comparative mean theta power variation from baseline between control and stressed animals immediately following closed arm entry (right). Data for open and closed arms are averages across animals of the normalized measure of theta power variation for all entries. **(B)** Comparative time evolution of mean average theta power variation from baseline during open arms risk-assessment preceding approach and avoidance actions for all stressed animals in vHPC, BLA, mPFC(PrL), and dHPC. Data was averaged across animals for each time point according to the subsequent action (approach or avoid). ^*^p < 0.05 for unpaired Wilcoxon rank sum test comparison of average theta power variation between control and stress groups and average theta power variation between activity preceding approach and avoidance actions. Error bars, ± sem.

Since we had previously shown (Jacinto et al., 2013) that higher theta power in the vHPC and BLA were correlated with avoidance of aversive locations of an environment, herein we first compared theta power immediately before (baseline; 0.5 s before risk-assessment period) and immediately after the start of open arm risk-assessment behavior (first 0.5 s). Stressed animals entering the open arms showed a much higher increase of mean vHPC and BLA theta power immediately following the start of the risk-assessment period than control animals, regardless of subsequent approach or avoidance actions (control vs. stress; vHPC: *p* < 0.05; BLA: *p* < 0.05; Figure 5A). dHPC theta power variation was of a similar nature, but lower magnitude (control vs. stress; *p* < 0.05, Figure 5A), whereas mPFC(PrL) theta power increased during the start of the risk-assessment period and such variation was similar in stress and control groups (Figure 5A).

Despite these differences, theta power variations in the vHPC and BLA of stressed animals during the risk-assessment period (up to 1.5 s in respect to the 0.5 s baseline that preceded the risk-assessment) mimicked those of controls: while a maintenance of high theta power preceded avoidance actions, approach actions were preceded by a significant decrease in power in both brain areas (*p* < 0.05 for each significant *post-hoc* pairwise comparison between approach and avoidance at 1.5 s; Figure 5B). There was also no significant difference between baseline theta power before approach or avoid actions; nor when the baseline of control and stressed animals was compared for approach and avoidance actions for all brain regions (Figure S2).

When analyzing theta power immediately following closed arm entry in respect to the baseline (the 0.5 s period immediately preceding the entry), there were no significant differences between controls and stressed animals in any of the recorded regions despite a clear trend for mPFC(PrL) theta power increase in both groups (Figure 5A). We also observed a decrease in mPFC(PrL) theta power before closed arm exit, as previously described (Adhikari et al., 2010), that was present in both control and stressed animals and occurred 1.0 to 1.5 s before the animal actually exited the closed arms (Figure S2). Despite a sharp transitory increase always observed during the exit or immediately after, overall mPFC(PrL) theta power was reduced outside the closed arms when compared with the power inside the arms previous to the described reduction anticipating the exit.

#### Increased theta coherence in BLA neuronal links is increased in stressed rats and relevant for anxiety

**Figure 6:**
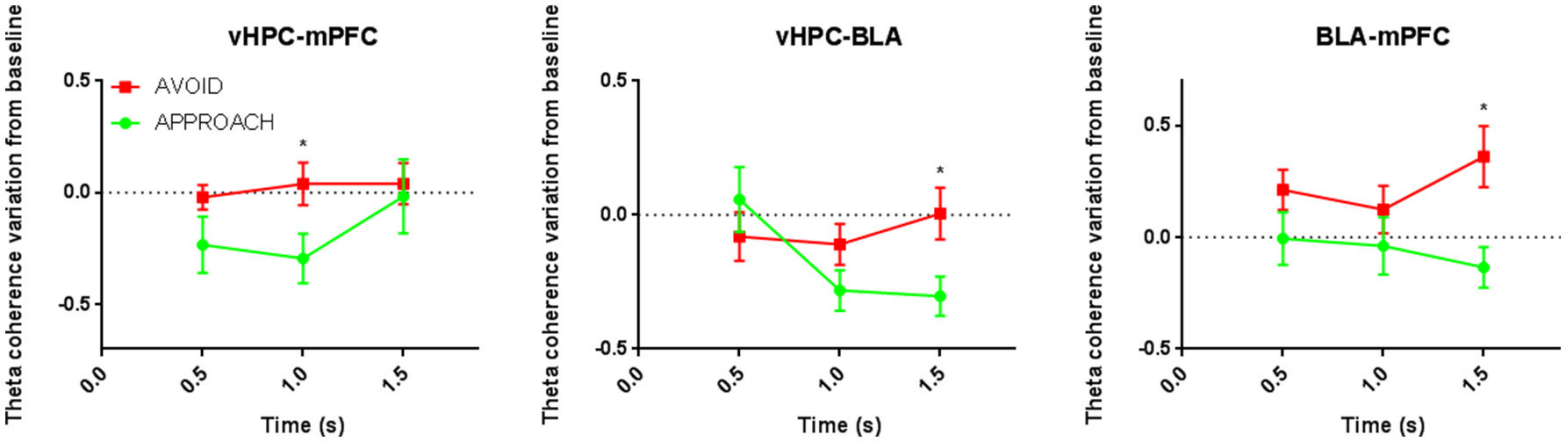
**Comparative time evolution of mean theta coherence variation from baseline during open arms risk-assessment preceding approach and avoidance actions for all stressed animals in brain areas' pairs vHPC-mPFC(PrL) (left), vHPC-BLA (middle), and BLA-mPFC(PrL) (right) brain areas' pairs**. Data was averaged across animals for each time point according to the subsequent action (approach or avoid ^*^p < 0.05 for unpaired Wilcoxon rank sum test comparison of average theta coherence variation between activity preceding approach and avoidance actions. Error bars, ± sem.

Similarly to controls, vHPC-mPFC(PrL) theta coherence variation during the risk-assessment period in stressed animals separated subsequent approach and avoidance actions (vHPC-mPFC(PrL) theta coherence variation: *p* < 0.05 for *post-hoc* pairwise comparison at 1.0 s; Figure 6). More importantly, in these animals, and contrary to controls, vHPC-BLA, and BLA-mPFC(PrL) theta coherence variations during risk-assessment were also correlated with the action of further exploring the open arms: while a decrease of vHPC-BLA coherence preceded approach actions, an increase of BLA-mPFC(PrL) coherence was correlated with subsequent avoidance actions (vHPC-BLA theta coherence variation: *p* < 0.05 at 1.0 and 1.5 s; Figure 6; BLA-mPFC(PrL) theta coherence variation: *p* < 0.05 at 1.5 s; Figure 6).

#### Locomotor activity cannot account for observed variations in theta power

While theta power has been seen to increase with running speed, most prominently in the septal pole of the HPC, theta frequency is usually more strongly related to speed (McFarland et al., 1975; Hinman et al., 2011). Thus, in the present study, a small, but significant, correlation of dHPC theta power with speed was observed (average *r* = 0.11 ± 0.03), but not of vHPC, BLA or mPFC(PrL) theta power (vHPC: −0.02 ± 0.01; BLA: 0.02 ± 0.01 mPFC(PrL): 0.01 ± 0.02). The absence of significant speed modulation, especially in the vHPC, BLA, and mPFC(PrL), reinforces the relevance of the above-described findings in the context of anxious behavior.

## Discussion

This study shows that theta power and coherence variations within the vHPC-AMY-mPFC(PrL) circuit are correlated with the outcome of risk-assessment behavior in the aversive region of the EPM, the open arms. In particular, variations of vHPC, BLA, and mPFC(PrL) theta power presented opposing patterns during the risk-assessment period before an approach or avoidance action took place. This was true for both control and stressed rats. Additionally, theta synchronization between the vHPC and mPFC(PrL), a connection critically involved in the anxiety response in the EPM (Adhikari et al., 2010), also presented opposing patterns during risk-assessment whether the future action was to approach or avoid the open arms. Opposing patterns correlated with the future action were also observed for vHPC-BLA and BLA-mPFC(PrL) theta synchronization but only for stressed rats.

The role of the hippocampus (HPC) in anxiety is not novel. In fact, in conflict contexts it has been claimed that the hippocampus can stop the motor program so that a risk-assessment period can take place. This period allows the gathering of more information from the environment so that the conflict can be resolved by re-directing behavior away from the most negative outcome (Gray and McNaughton, 2003). The present observations lend further support to this hypothesis by showing that when assessing the risk of entering the open arms of the EPM, where the animal faces a conflict between exploring the unknown and elevated arm or retreating to the “safer” closed arm, vHPC's theta power is correlated with state anxiety and discriminates between subsequent exploration of (approach) and retreat from (avoidance) the open arms. Interestingly, a recent fMRI study in humans also identified a causal role for the anterior hippocampus, the human homolog of the rodent VHPC, in the approach-avoidance conflict resolution (Bach et al., 2014). These observations are in line with, and extend, our previous findings that theta activity of the ventral portion of the HPC is correlated with exploratory behavior in an anxiety context (Jacinto et al., 2013) in a link which may be mediated by downstream brain areas to which the HPC is strongly connected. Indeed, we also reveal that the strong connectivity of the vHPC, especially in the theta oscillations range, with other brain areas like the AMY and the mPFC(PrL) may also provide clues on how the observed vHPC activity may contribute to the decision of further exploring or abandoning the open arms of the EPM.

The AMY, in particular the BLA, is strongly interconnected with the vHPC (Pitkänen et al., 2000) and is profoundly implicated in the processing of threatening stimulus and defensive behaviors including in an anxiogenic context (Phelps and LeDoux, 2005; Tye et al., 2011; Wang et al., 2011; Felix-Ortiz et al., 2013). As a result, co-activation of the vHPC and BLA in an anxiogenic situation can be expected (Felix-Ortiz et al., 2013) as anxious exploration modulated by the hippocampus also requires increased arousal and readiness of the fight-flight system in case any potential threat materializes (Gray and McNaughton, 2003; Jacinto et al., 2013). It is, thus, plausible that an overactivation of the HPC and AMY may signal the negative valence of a possible threat stimulus and that the outcome of the animals' decision to fully enter or avoid the open arms depends in part on the modulation of activity in this limbic link, as supported by the present data. Our results suggest that the modulation of synchronous activation of the vHPC-BLA, in the theta range, occurring within the open arms' risk-assessment period correlates with the subsequent action of further exploring (if activity decreases) or abandoning (if activity increases) the open arm. Whether these variations are only neuronal hallmarks of the anxiety-driven risk-assessment in the brain areas that modulate anxiety or are themselves regulating behavior is an open question; theta disruption studies are needed to clarify this issue. Nevertheless, theta changes have been previously shown to be causally related with changes in behavior (Turnbull et al., 1994; McNaughton et al., 2007; Shirvalkar et al., 2010). Moreover, inactivating or lesioning the vHPC or BLA reduces anxious-like behavior (Adamec et al., 1999; Pentkowski et al., 2006) and an optogenetic study attributed a causal role to the vHPC-BLA link in the modulation of anxiety behavior (Felix-Ortiz et al., 2013).

The mPFC, and its interplay with the HPC, has also been implicated in anxiety (Lacroix et al., 2000). Communication via theta oscillations between vHPC and mPFC have been implicated not only in learning actions (Benchenane et al., 2010) but also in the modulation of anxiety-like behavior (Adhikari et al., 2010; Padilla-Coreano et al., 2016). More precisely, increased vHPC-mPFC theta synchrony has been correlated with increased avoidance of the EPM's open arms (Adhikari et al., 2010; Padilla-Coreano et al., 2016). In accordance with this finding, we have also observed that the decision to abandon the open arms after risk-assessment was correlated with an increase in vHPC-mPFC(PrL) theta coherence while the decision to further explore them was correlated with the opposite modulation. Thus, the present observation of distinct power variation in the vHPC during risk-assessment is likely to be signaled to the mPFC (Padilla-Coreano et al., 2016). It is possible that the vHPC signals state anxiety and communicates this state to other brain regions (e.g., AMY and mPFC) to re-direct behavior accordingly—although inputs from the BLA to the vHPC and from the mPFC to the AMY have also been shown to be important in the modulation of anxiety in certain contexts (Felix-Ortiz et al., 2013; Adhikari et al., 2015).

The decision to further explore or abandon the open arms was correlated with mean vHPC, but not dHPC, theta activity. This intra-hippocampal specificity is not surprising, given the functional dissociation attributed to the region, namely concerning anxiety-like behavior (Bannerman et al., 2003). Yet, it should be noted that the dorsal and ventral regions of the HPC are interconnected and theta waves may travel along its axis (Patel et al., 2012); in fact, there is at least one study reporting that the magnitude of theta oscillations recorded from the dHPC in serotonin 1A receptor-deficient mice, a strain which displays increased anxiety-like behavior, increased in the EPM in respect to a familiar environment (Gordon et al., 2005).

Stressed animals tend to display increased anxiety-like behavior in the EPM, avoiding the open arms more frequently than controls (Pêgo et al., 2008), as confirmed herein. Interestingly, this stress-induced anxiety status was associated with increased theta power in the vHPC and BLA during risk-assessment of the open arms. Overactivation of the vHPC and BLA by stress has been previously described in studies on brain slices (Rainnie et al., 2004; Maggio and Segal, 2009), anesthetized rats (Kavushansky and Richter-Levin, 2006; Oliveira et al., 2013; Pinto et al., 2015) and freely moving rats (Jacinto et al., 2013); this correlation may either be an expression of increased anxiety or, more appealingly, the precise signaling that leads stressed animals to attribute a higher negative valence to the open arms than controls. Interestingly, and similar to the observed variations in controls, theta power variations in the vHPC and BLA of stressed animals during open arm risk-assessment were also a predictor of subsequent actions in the EPM. This observation confirms that the modulation of theta power in these brain regions is strongly correlated with the subsequent decision of further exploration of the most anxiogenic portion of the EPM, the open arms, and may in fact be a relevant signal for the decision-making process in this conflict context. Taking it one step further, this also suggests that theta modulation in these brain areas may be a relevant therapeutic target for anxiety (and indeed anxiolytic drugs of all know classes affect theta oscillations in the hippocampus (McNaughton et al., 2007). vHPC-mPFC(PrL) synchrony during risk-assessment was also correlated with the subsequent approach or avoidance decision in stressed animals further reinforcing the role of this link in anxiety-like behavior. However, unlike in control animals, vHPC-BLA and BLA-mPFC(PrL) theta coherence variations during the same period were also able to differentiate subsequent approach or avoidance actions, with the absence of decrease in BLA-vHPC and BLA-mPFC(PrL) theta coherence during open arm risk-assessment correlating with the decision to abandon the open arms. This observation is in accordance with the well-known pivotal role of stress upon AMY activity (Vyas et al., 2002; Roozendaal et al., 2009) and suggests that the overactivation of this area, and the ensuing increased activity in its connections with the vHPC and the mPFC(PrL), might be a critical factor in the manifestation of stress-induced anxiety-like behavior. This also re-enforces previous studies reporting that the functional connectivity, including in the theta range, between the hippocampus and amygdala is enhanced by stress (Maggio and Segal, 2012; Ghosh et al., 2013; Jacinto et al., 2013) and that BLA-mPFC(PrL) theta synchrony increases with anxiety (Jacinto et al., 2013; Likhtik et al., 2014).

In conclusion, we show for the first time that power variations in the vHPC-BLA-mPFC(PrL) circuit during the risk-assessment exploration of the EPM open arms are correlated with the animal's subsequent action to approach or avoid the open arm. We show that theta power decreases in the vHPC and BLA and increases in the mPFC(PrL) during risk assessment when an approach action follows; while the opposite variations occur preceding a retreat action. In addition, we also reveal that the networks involved in the resolution of this conflict are different in control animals and in a model of stress-induced anxiety: while in controls the further exploration of the open arms appears to be correlated with vHPC-mPFC(PrL) coherence only, stressed animals' decisions seems to be modulated by an increased BLA activation, with the consequent enhancement of BLA-vHPC and BLA-mPFC(PrL) links besides the vHPC-mPFC(PrL) connection. These observations reinforce the view of the vHPC-BLA-mPFC(PrL) network as a critical circuit in physiological and pathological conditions.

## Methods

### Animals

A total of 10 Male Wistar-Han rats (Charles River laboratories, Barcelona, Spain), weighing 300–350 g and aged 12 weeks (at the time of surgery) were used in this study. Animals were single-housed under the following laboratory conditions: room temperature 22°C, relative humidity of 55%, 12 h light cycle beginning at 8 a. m., food and water *ad libitum*. Experiments were conducted in accordance with European Union Directive 2016/63/EU and the Portuguese regulations and laws on the protection of animals used for scientific purposes of the Ministry for Agriculture, Rural Development and Fishing. This study was approved by the Portuguese Veterinary General Direction, Direcção Geral de Alimentação e Veterinária (DGAV).

### Surgery

Following a period of 2 weeks of handling for at least once a day, animals were subjected to a surgery for implantation of chronic single-wire electrodes. Electrodes were assembled in-house from formvar insulated nichrome single wires (Science Products GmbH, Hofheim, Germany), 50 μm inner diameter, and golden Mill-Max receptacles (Mill-Max Mfg. Corp., Oyster Bay, NY, USA). Animals were kept under anesthesia during the whole procedure with a gaseous mixture of 2–4% sevoflurane in 100% oxygen. Electrodes were implanted, through skull burr-holes, and targeted the mid-ventral portion of the pre-limbic area of the prefrontal cortex (3.3 anterior, −0.8 lateral and 4.0 depth), the dorsal portion of the hippocampus (3.9 posterior, −2.2 lateral and 2.4 depth), the ventral portion of the hippocampus (4.8 posterior, −4.8 lateral and 8.4 depth) and the basolateral amygdala (2.4 posterior, −4.9 lateral and 8.6 depth). A stainless-steel screw electrode over the cerebellum (10.5 posterior, 0.0 lateral) served as ground. Distances are in mm from bregma. All electrodes were cemented directly to the skull and connected to a Mill-Max connector. The final assembly was cemented with dental acrylic resin (GC America Inc., Alsip, IL, USA), with four additional skull screws serving as anchors. Animals were allowed to recover for 15 days.

Following the recovery period, animals were familiarized with the recording room and tethering procedure in 20 min familiarization sessions during 5 days.

### Stress protocol

To confirm the validity of the analysis in control rats and assess how stress could have a differential effect on the vHPC-BLA-mPFC(PrL) circuit 5 rats were exposed to a chronic unpredictable stress (CUS) protocol, described elsewhere (Cerqueira et al., 2007), for 21 days. Importantly, exposure to this CUS protocol is known to induce anxiety-like behavior (Pêgo et al., 2008). Briefly, stressed animals were exposed to a daily stressor (up to 1 h). In order to avoid adaptation the stressor applied was different every day and presented at a different hour of the day. Four different stressors were used: restraint, noise, shaking and cold air stream. The stress protocol started after the familiarization period with the recording room and procedures. All stressors were applied in a separate experimental room from where the animals of both groups were housed. Control group animals (*n* = 5) were handled for the same time during the same period.

On the day following the end of the stress protocol blood samples were drawn from all animals (stress and control groups) via tail venipuncture for serum corticosterone levels assessment. Blood samples were collected in the morning. The samples were centrifuged at 13,000 rpm for 10 min. Serum was extracted and stored at −80°C for posterior analysis. Serum corticosterone levels were measured using 125I radioimmunoassay (RIA) kits (MP Biomedicals, Inc, Orangeburg, NY, USA). Reduced or slow body weight gain has also been associated with the efficacy of stress protocols (Pêgo et al., 2008); therefore, body weights of all animals were recorded on a weekly basis and body weight gain between the first and last days of the stress protocol was calculated.

### Elevated-plus maze test

Following 1 day of rest after blood collection, animals from both groups were exposed to the Elevated-Plus Maze (EPM) test with a duration of 5 min. The EPM is a validated test to assess anxiety-like behavior in rodents and the protocol has been described elsewhere (Sousa et al., 2006; Walf and Frye, 2007).

### Data acquisition

Signals were acquired during EPM performance in single-ended non-referenced mode using the dacqUSB system (Axona Ltd., London, UK) at 24 kHz. Field potential signals were amplified and low-pass filtered with a 600 Hz cut-off frequency. A 50 Hz notch filter was applied in all recordings. Position coordinates were also acquired (20 Hz) with an integrated video-tracking system from an infra-red LED on the headstage connected to the animal's headmount.

### Data analysis

Data was imported into Matlab (Mathworks, Natick, MA, USA) and analyzed with custom-written code and Chronux toolbox (http://chronux.org/) (Mitra and Bokil, 2008). Data was first downsampled to 1.2 kHz and detrended using the function *locdetrend* from the Chronux toolbox (*window size* 0.5 s; *step* 0.1 s). Time instants of open and closed arms entries were automatically obtained via position tracking data in matlab. All animals performed open arm risk-assessment entries at least five times, a pre-requisite we had set for further analysis. Transition data contaminated with saturation or movement artifacts were removed from posterior analysis. Theta power estimates were calculated with a multitaper method using Chronux. Half-second windows with no overlap were used for the analysis of open and closed arms' transitions. The time-bandwidth product (TW) was chosen as 3 and the number of tapers (K) was 5. Frequency resolution was chosen to be 0.6 Hz. Theta spectral coherence between all brain regions was calculated as the cross-spectrum of each LFP pair normalized by their auto-spectra. The spectrum estimates were obtained by the multitaper method for the same windows used in the power estimates using similar multitaper parameters. Total theta power on each window was obtained by the summation of spectral power estimates of all frequencies in the 5–12 Hz band while theta coherence was averaged for all estimates in the same frequency band. Theta power and theta coherence during risk-assessment periods were analyzed in 0.5 s windows up to 1.5 s after the beginning of the open arm risk-assessment entry with respect to a 0.5 s baseline period prior to the entry. Theta power and theta coherence variations for each time bin during the risk-assessment period were given by the ratio of the theta power or coherence estimate in the analyzed time bin minus the estimate in the baseline bin by the estimate in the analyzed time bin. This normalization procedure was calculated for each animal and then averaged across animals within each group for each time bin. The calculated normalized measure, for both theta power and theta coherence, is positive if power or coherence increases in respect to the baseline period, negative if it decreases and takes a value of zero if unchanged.

Exemplificative average spectrograms (Figure 2) for the activity preceding approach and avoidance actions for one animal were calculated for 0.5 s windows (with 90% overlap) for the time period of the theta power variation analysis (from 0.5 s before open arm risk-assessment entry to 1.5 s after the entry). Exemplificative spectrograms for mPFC(PrL) theta power proceeding and following closed arm exits (Figure S3) were calculated in the same way but spanning a longer time period (from 3.0 s prior to the exit up to 3.0 s following the exit).

To test if the variations in theta power observed could be accounted for by speed modulation, the total time of each recording for each brain area was divided in 0.5 s non-overlapping segments and mean theta power and mean speed were calculated for each segment. Speed was calculated as the distance between two consecutive tracking positions obtained by the video-tracking system during test performance; and mean segment speed was obtained by averaging all speed values within each segment. Pearson correlation coefficients between speed and theta power were averaged across animals for the same brain regions.

### Histology

To confirm the position of the electrodes, at the end of the experimental period, all animals were deeply anesthetized with pentobarbital (100 mg/Kg). An electrolytic lesion was done by passing current through all the electrodes. The animals were then perfused transcardially with fixative (4% paraformaldehyde). The brains were removed and placed in fixative solution. After further fixation the brains were coronally sectioned in 45 μm slices, collected on non-coated glass slides, stained with Giemsa and mounted with Entellan-New (Merck, Darmstad, Germany). Electrode tip position was determined by microscopic observation of the slides.

### Statistics

Two-way analysis of variance (two-way ANOVA) was used to assess significant interactions between evaluated time-points (0.5; 1.0 and 1.5 s) during risk-assessment and subsequent actions (approach vs. avoid) as well as their effect on the power and coherence measures for each brain are. There were no significant interactions between timepoints and actions. Only actions showed significant simple main effects on theta power and coherence measures for. ANOVA was followed by *post-hoc* pairwise comparisons using Bonferroni correction between approach and avoid actions for each time-point separately. Comparisons of two groups (corticosterone levels, weight gain and EPM performance between stress and control groups) were done by Welch's *t*-test. Results are expressed as mean ± standard error of the mean (sem).

## Author contributions

LJ, JC, and NS designed the experiment. LJ acquired and analyzed all data. JC and NS supervised the experiment. LJ and NS wrote the paper. All authors contributed to the final/submitted version of the work.

## Funding

LJ was supported by fellowships: SFRH/BD/40459/2007 from the Portuguese Foundation for Science and Technology (FCT); UMINHO/BPD/27/2013 funded by CCDR-N and Programa Operacional Região Norte (ON.2) from QREN/FEDER; and UMINHO/BPD/63/2015 from Fundação Calouste Gulbenkian funded project (contract grant number P-139977). Financial support for this work was provided by FEDER funds through the Operational Programme Competitiveness Factors - COMPETE and National Funds through FCT under projects FCOMP-01-0124-FEDER-021145, FCT/PTDC/SAU-ENB/118383/2010, FCOMP-01-0124-FEDER-022674 and POCI-01-0145-FEDER-007038; and by the project NORTE-01-0145-FEDER-000013, supported by Norte Portugal Regional Operational Programme (NORTE 2020), under the PORTUGAL 2020 Partnership Agreement, through the European Regional Development Fund (ERDF).

### Conflict of interest statement

The authors declare that the research was conducted in the absence of any commercial or financial relationships that could be construed as a potential conflict of interest.
