# Supplementary material for: Patterns of Theta Activity in Limbic Anxiety Circuit Preceding Exploratory Behavior in Approach-Avoidance Conflict
Source: Front Behav Neurosci. 2016 Sep 22;10:171. doi: 10.3389/fnbeh.2016.00171 (PMC5031779; doi:10.3389/fnbeh.2016.00171)
Supplement: Supplementary file 1 [file Image1.PDF]

FIGURE S1

A

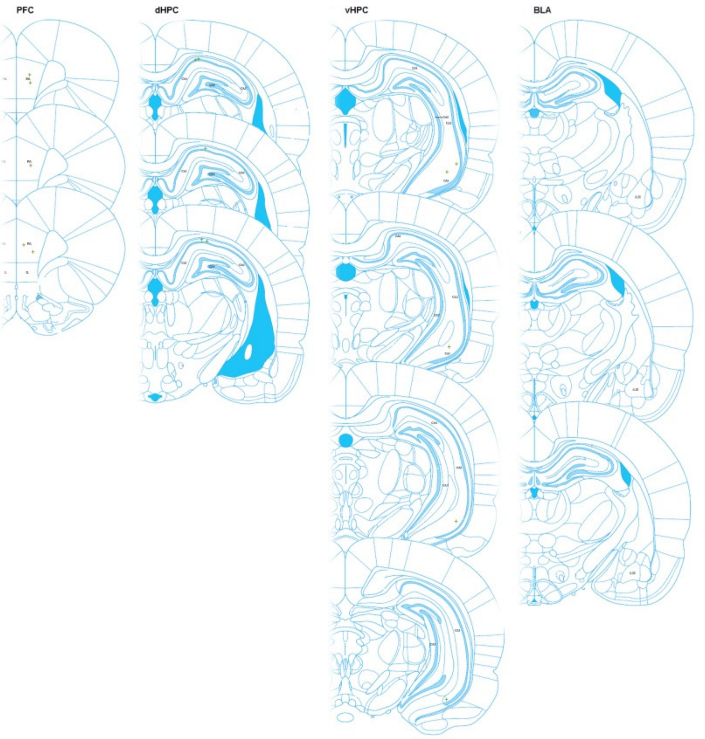

B

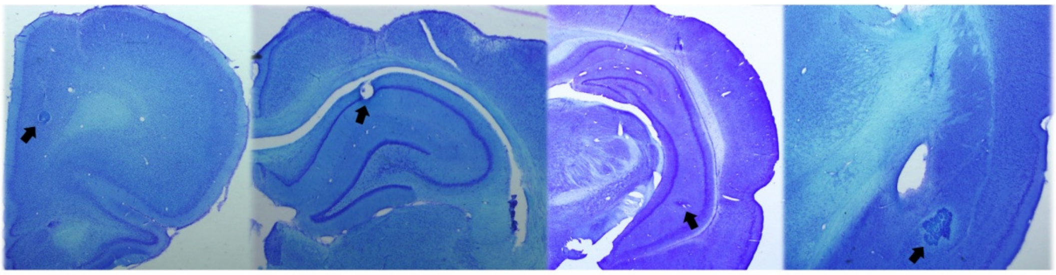

Figure S1. (A) Diagrammatic coronal sections of mPFC (left), dHPC (center left), vHPC (center right) and BLA (right). Sections are arranged from most anterior (top) to most posterior (bottom). Electrolytic lesion sites are marked as crosses. Electrode positions are shown as green. PL: prelimbic area of PFC; IL: infralimbic area of mPFC. Diagrammatic coronal sections are adapted from (Paxinos and Watson, 2006). (B) Representative histological sections showing electrolytic lesions in the PL of the mPFC (left), dHPC (center left), vHPC (center right) and BLA (right). Black arrows mark electrode position.

SUPPLEMENTAL REFERENCE: Paxinos, G., and Watson, C. (2006). *The Rat Brain in Stereotaxic Coordinates, 6th Edn.* London: Elsevier.
