# Supplementary material for: Patterns of Theta Activity in Limbic Anxiety Circuit Preceding Exploratory Behavior in Approach-Avoidance Conflict
Source: Front Behav Neurosci. 2016 Sep 22;10:171. doi: 10.3389/fnbeh.2016.00171 (PMC5031779; doi:10.3389/fnbeh.2016.00171)
Supplement: Supplementary file 2 [file Image2.PDF]

FIGURE S2

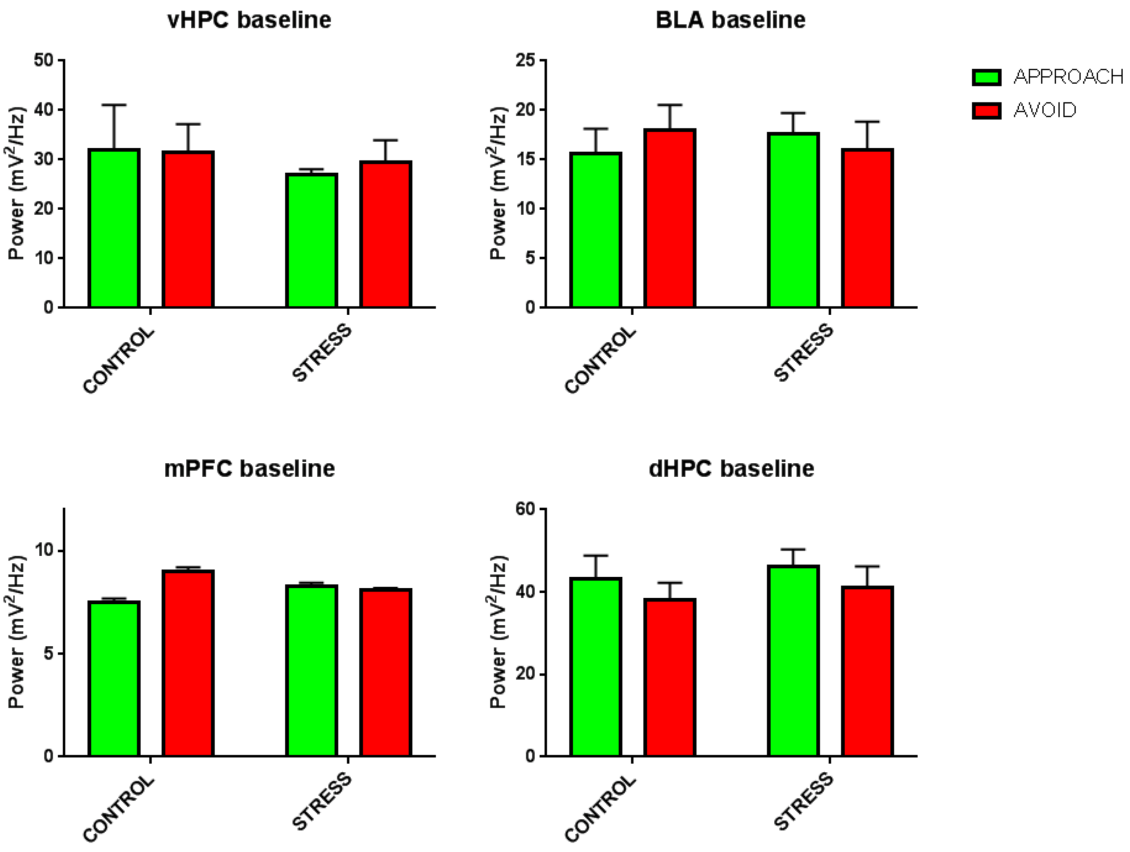

Figure S2. Averaged baseline theta power for control and stressed animals preceding approach or avoidance actions for vHPC (top left), BLA (top right), mPFC(PrL) (bottom left) and dHPC (bottom right).
