# Supplementary material for: Patterns of Theta Activity in Limbic Anxiety Circuit Preceding Exploratory Behavior in Approach-Avoidance Conflict
Source: Front Behav Neurosci. 2016 Sep 22;10:171. doi: 10.3389/fnbeh.2016.00171 (PMC5031779; doi:10.3389/fnbeh.2016.00171)
Supplement: Supplementary file 3 [file Image3.PDF]

FIGURE S3

A

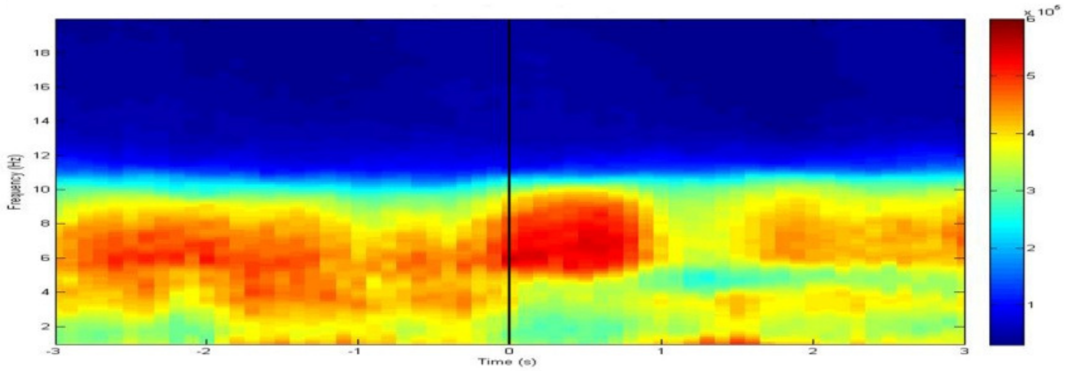

B

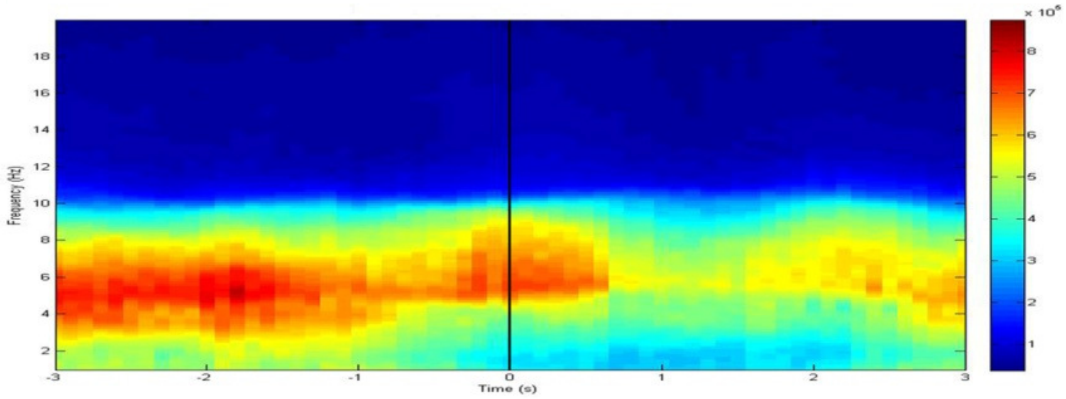

Figure S3. mPFC theta power spectrograms for closed arms' exits for one control (A) and one stressed (B) animal. Spectrogram for each animal is an average of all closed arm exits performed during the EPM test.
